# 

**Published:** 1881-03

**Authors:** 


					﻿MARCH, 1881.
TWENTY-EIGHTH YEAR.
ELAJLJLi’S
OF
mILiOmi iJShja3	Jliartfrl a&i
E. H. GIBBS, A.M., M.D., Editor,
No. 141 EIGHTH STREET (Near Broadway), NEW YORK.
TERMS: $2 a Year, or $1 for Six Months. Single nnmher, 20 cis.
				

